# Anatomical description of neornithine stomach with implications on neornithine stomach morphology

**DOI:** 10.1111/joa.14123

**Published:** 2024-08-17

**Authors:** Ryuji Takasaki, Yoshitsugu Kobayashi

**Affiliations:** ^1^ Department of Natural History and Planetary Sciences Hokkaido University Hokkaido Japan; ^2^ Department of Ecology & Evolutionary Biology University of Toronto Toronto Ontario Canada; ^3^ Faculty of Biosphere‐Geosphere Science Okayama University of Science Okayama Japan; ^4^ Hokkaido University Museum Hokkaido University Hokkaido Japan

**Keywords:** diet, neornithine, stomach

## Abstract

Neornithines, the most diversified extant tetrapods, have been a classic example for understanding form–function relationships, particularly in the context of the interaction between dietary ecology and neornithine phenotypic evolution. While the previous studies have primarily focused on beak morphology, the significance of the neornithine stomach, which serves as a functional analog of mammalian dentition, is expected to play an important role as well. However, current knowledge on neornithine stomachs is predominantly biased toward poultry and birds of prey, leading to a significant underappreciation of its impact on macroevolution. Here, we provide detailed descriptions of neornithine stomachs represented by 115 species of major orders and test if and how neornithine stomachs are related to their dietary ecology. We identified four morphotypes among neornithine stomachs, which are strongly constrained phylogenetically. While the neornithine diet demonstrates strong associations with stomach morphotypes, the associations are small or absent when accounting for the phylogeny in statistical evaluations. Similarly, the neornithine diet has negligible effects on their ventriculus mass under the analyses with phylogenetic correction. The results resemble a recent finding that a neornithine diet has no effect on intestine length when accounting for phylogeny, but rather flight performance does. Thus, the present study further supports the previous findings that dietary specialization in neornithine birds closely follows phylogeny, making functional convergence across taxa difficult to detect.

## Graphical abstract

The representatives of the four neornithine stomach morphotypes proposed in this study.

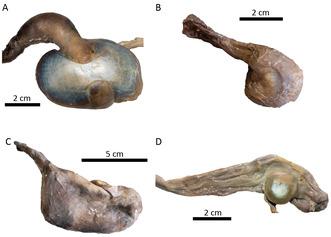

## 1 INTRODUCTION

The dietary ecology of living organisms is closely linked to individual fitness (Grant & Grant, 2006; Nicolson & Fleming, 2003) and is subject to intense selection pressure. Consequently, animal evolutionary traits have been shaped by multiple convergences and trade‐offs, largely reflecting dietary strategies (Barnagaud et al., 2019; Bels & Herrel, 2019). Neornithines, comprising over 10,000 species today, inhabit virtually all habitable environments (Lovette & Fitzpatrick, 2016) and serve as a classic example of such dietary adaptations (Grant & Grant, 1993; Jonsson et al., 2012; Lovette et al., 2002). Previous studies have extensively investigated the relationship between neornithine diet and skull morphology, primarily focusing on beak morphology, and some supported the form–functional relationships (e.g., Olsen, 2017; Soons et al., 2015) while others found little association (e.g., Bright et al., 2016; Felice et al., 2019; Navalon et al., 2019). Neornithines further exhibit sets of highly specialized digestive organs including crop for food storage (Kierończyk et al., 2016) and caeca that perform retrograde urine transport that enhances uric acid fermentation (Björnhag, 1989; Frei et al., 2017), while the intestine length was shown to correlate more strongly to flight mode than to diet (Duque‐Correa et al., 2022).

The neornithine stomach has also experienced a significant degree of adaptation, including a significantly high amount of myoglobin in herbivorous taxa (Enoki & Morimoto, 2000). The neornithine stomachs also exhibit morphological variations, which have traditionally been considered to be associated with dietary ecology (Ziswiler & Farner, 1972). The neornithine stomach consists of two distinct chambers: the proventriculus, primarily serving for mucus secretion (HCl and pepsin), and the ventriculus, also known as the gizzard, which functions as the primary site for mechanical processing (Moore, 1999). Previous studies have shown that carnivorous and piscivorous taxa generally possess a large proventriculus and a less muscular ventriculus, functioning primarily as food storage. In contrast, herbivorous taxa typically have a small proventriculus and a highly muscular ventriculus for the mechanical processing of ingesta (Owen, 1866; Pernkopf, 1929; Ziswiler & Farner, 1972). However, these findings are biased toward poultry and birds of prey, and no statistical evaluation has been performed as far as the authors are aware. Further, later studies suggest much larger variations in neornithine stomach morphology than the binary herbivorous type against carnivorous type (Degen et al., 1994; Desselberger, 1931; Jackson & Place, 1990).

The aim of this study is to (1) develop the criteria that categorize neornithine stomach morphotype and then (2) investigate if neornithine stomach morphology corroborates with their dietary ecology. We evaluate neornithine stomachs using stomach morphotype as a qualitative proxy and gizzard muscularity (Takasaki & Kobayashi, 2020a) as a quantitative proxy. We then infer their interactions with a diet under a phylogenetic framework.

## 2 METHODS

### 2.1 Stomach variations

Stomachs of both wild and captive birds were dissected to observe stomach morphological variations. All specimens used in this study are collections of the Hokkaido University Museum (HoUMVC) and the Botanic Garden of Hokkaido University (HUNHM). The samples were either fixed and stored in 10% neutral formalin or fixed with 10% neutral formalin and then stored in 70% ethanol and stored in the institutes. The final dataset (Data S1) comprises 368 individuals from 115 species, covering major neornithine orders. While neornithine stomach shows a considerable degree of intraspecific variations, often associated with diet and season (e.g., Dekinga et al., 2001; Takasaki & Kobayashi, 2020a), the current dataset includes taxa with only one sample to maximize the taxonomic sampling.

Neornithine stomachs were evaluated using both qualitative and quantitative proxies. The qualitative proxy builds upon previous studies that identified two extremities in neornithine stomachs (Denbow, 2015; Klasing, 1998), incorporating variations in stomach morphology. Ventriculus and body masses were measured from each individual. As the qualitative proxy, neornithine stomachs were categorized into four morphotypes based on combinations of seven morphological characters (see the Result section). The validity of this categorization was tested by calculating the Gower dissimilarity matrix, followed by hierarchical cluster analysis using R package *cluster* version 2.1.3 (Maechler et al., 2021). For the taxa showing intraspecific variations, the characters were scored as unknown. In contrast to Takasaki and Kobayashi (2020a), which used the ratios of ventriculus mass to body mass, this study uses residuals of the two variables in order to incorporate allometry. Since the ventriculus is the only site for mechanical processing throughout the neornithine digestive tract (Moore, 1999), gizzard muscularity contributes to a key functional role in neornithine dietary ecology. Additionally, due to limited space within the abdominal cavity, ventriculus muscle mass is expected to be in a trade‐off relationship with the storage capacity. Thus, high gizzard muscularity represents high mechanical processing ability, while low gizzard muscularity is indicative of a larger storage capacity. Dietary ecologies were brought from Wilman et al. (2014), following their labels. This study uses both the categorical (vertebrate, invertebrate, plant + seed, omnivore, fruit + nectar) and diet proportions (proportion of invertebrates, %Inv; proportion of endothermic vertebrates, %Vend; proportion of ectothermic vertebrates, %Vect; proportion of fish, %Vfish; proportion of unknown vertebrates, %Vunk; proportion of scavenging, %Scav; proportion of fruit, %Fruit; proportion of nectar, %Nect; proportion of seed, %Seed; proportion of plant, %Plant) to test if diet affected neornithine stomach morphology.

### 2.2 Stomach evolution

Fisher's exact test was performed to test the associations between neornithine dietary categories against stomach morphotypes. Pairwise *t*‐tests and phylogenetic analysis of variance (ANOVA), both adjusted using the Bonferroni correction, were performed to test the relationships between diet proportions and stomach morphotypes with and without phylogenetic correction. Phylogenetic ANOVA was performed using *phytools* version 0.7‐70 (Revell, 2012). For the comparisons among different categories, morphotype A and fruit + nectar feeders were used as the reference category. Phylogenetic signal estimations (using lambda and kappa as the signals) and model fitting were performed using *geiger* version 2.0.7 (Pennell et al., 2014). Effects of stomach morphotypes, diet categories, and diet proportions were tested using generalized least squares (GLS) and phylogenetic generalized least squares (PGLS), the latter using *caper*.

The analyses under phylogenetic frameworks were conducted across 100 phylogenetic frameworks (Data S1) obtained from BirdTree.org (Jetz et al., 2012; Rubolini et al., 2015), utilizing phylogenetic reconstructions based on Ericson backbone (Ericson et al., 2006). To account for phylogenetic uncertainties, all analyses were repeated for the 100 trees (Data S2), and the median values are presented here. The significance level was set at 0.05. All statistical analyses were performed using R version 4.0.3 (R Core Team, 2020). The results of the statistical analyses were visualized using basic R functions and R packages *phytools* version 0.7‐70 (Revell, 2012). The R script used for the analyses is provided as Data S3.

## 3 RESULTS

**FIGURE 1:**
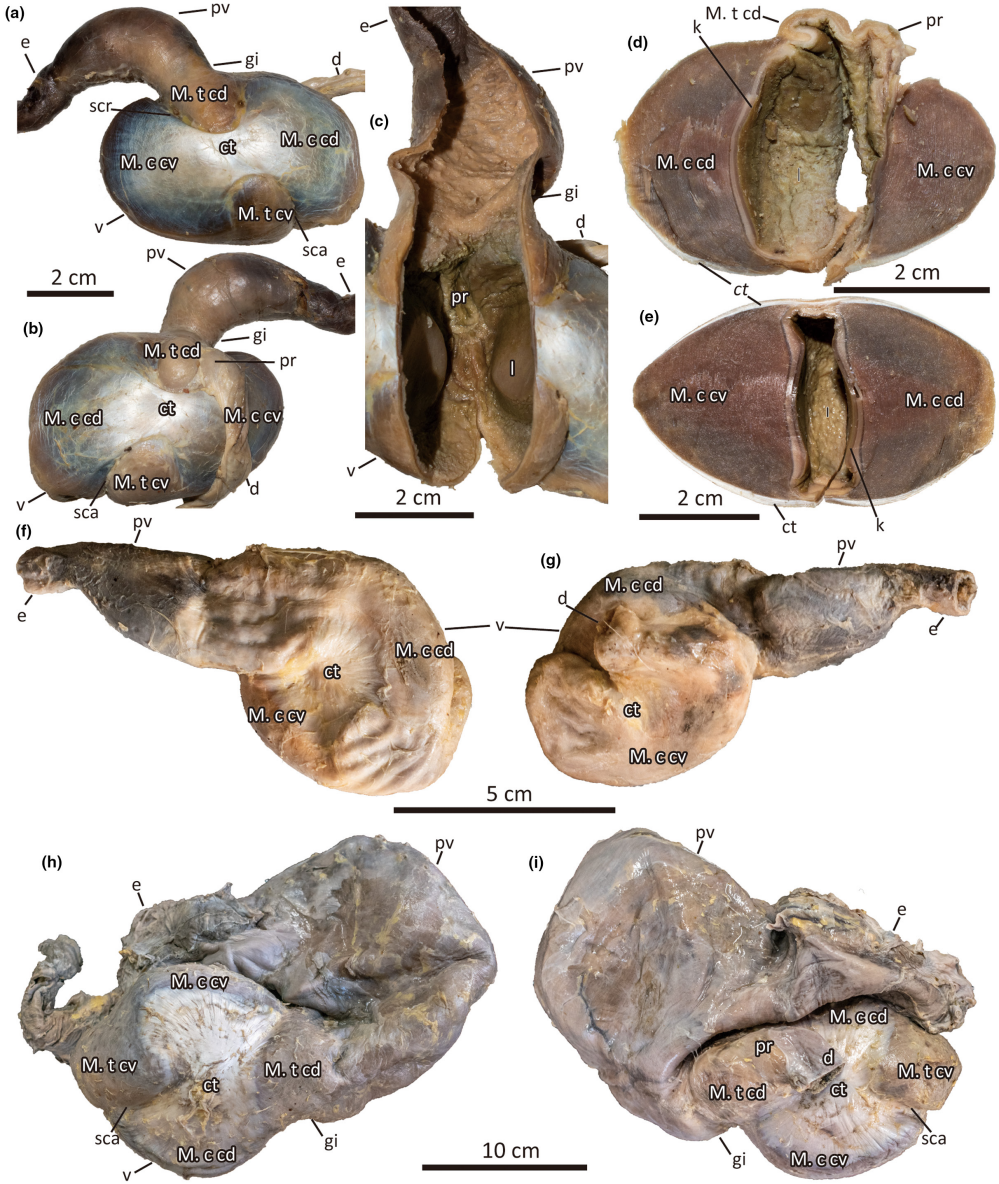
Representatives of type A stomachs. The stomach of *Anas acuta* in (a) left and (b) right lateral views. (c) The internal cavity of the *Anas acuta* stomach. (d) Transverse section of the ventriculus at the level of the pyloric region. Note that the pyloric region does not form a distinct chamber but is a groove in between two large gastric folds. (e) Transverse section of the ventriculus at its maximum width. (f–g) Stomach of *Dromaius novaehollandiae* in (f) left and (g) right lateral views. The stomach of *Struthio camelus* in (h) left and (i) right lateral views. ct, centrum tendineum; d, duodenum; e, esophagus; gi, gastric isthmus; k, koilin layer; l, lumen; M. c cd, M. crassus caudodorsalis; M. c cv, M. crassus cranioventralis; M. t cd, M. tenuis craniodorsalis; M. t. cv, M. tenuis cranioventralis; pr, pyloric region; pv, proventriculus; sca, sulcus caudalis; scr, sulcus cranialis; v, ventriculus.

**FIGURE 2:**
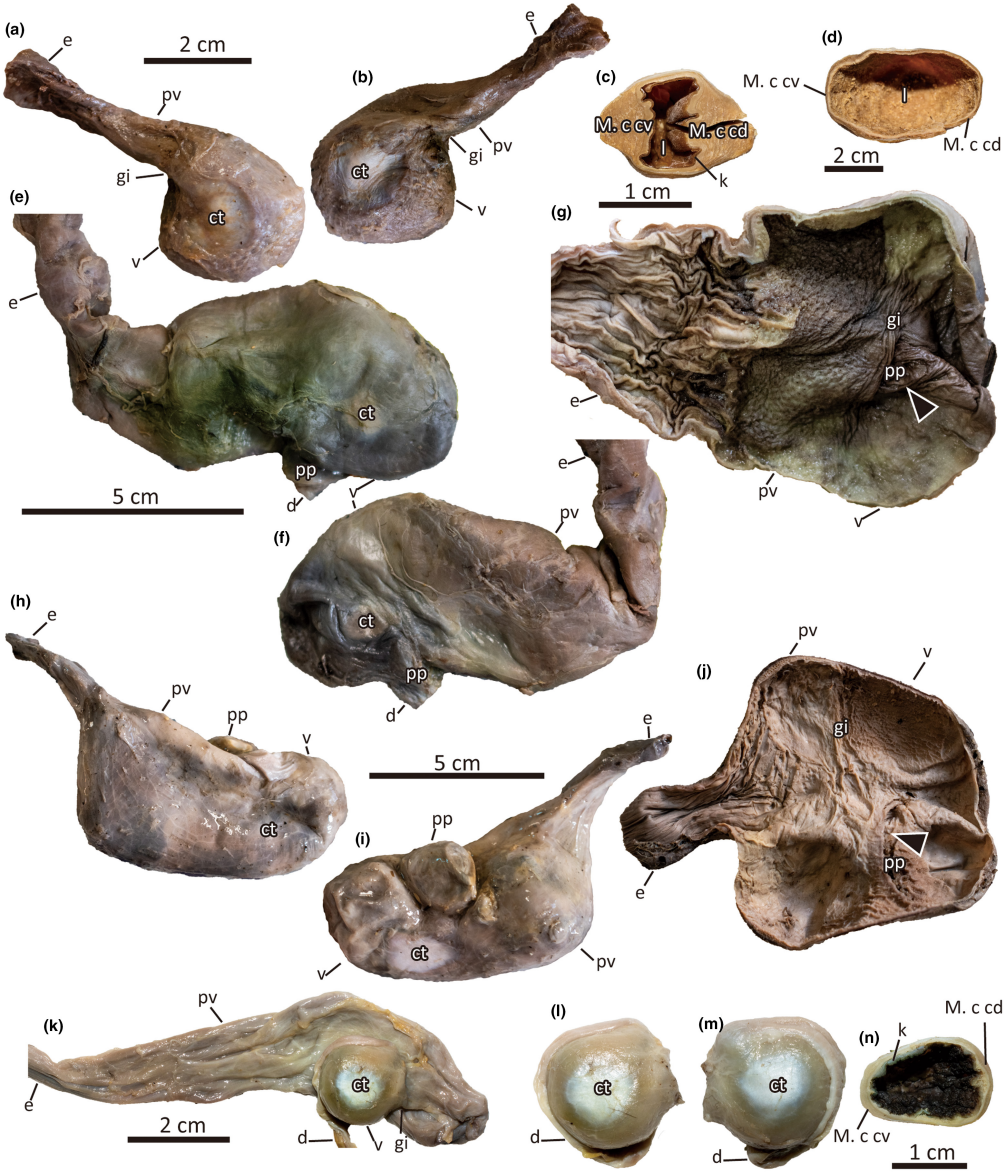
Morphotype B (a–d). The stomach of *Strix uralensis* in the left (a) and right (b) lateral views. Transverse sections of *Picus awokera* (c) and *Milvus migrans* (d) ventriculus. Morphotype C (e–j). The stomach of *Phalacrocorax carbo* in (e) left and (f) right lateral views. (g) The internal cavity of the *Phalacrocorax carbo* stomach. The stomach of *Ardea cinerea* in (h) left and (i) right lateral views. (j) The internal cavity of the *Ardea cinerea* stomach. Morphotype D stomach *Puffinus tenuirostris* (k–n). Left lateral view of the whole stomach (k). *Puffinus tenuirostris* ventriculus in left lateral (l), right lateral (m), and transverse (n) views. ct, centrum tendineum; d, duodenum; e, esophagus; gi, gastric isthmus; k, koilin layer; l, lumen; M. c cd, M. crassus caudodorsalis; M. c cv, M. crassus cranioventralis; M. t cd, M. tenuis craniodorsalis; M. t. cv, M. tenuis cranioventralis; pr, proventriculus; pv, proventriculus; sca, sulcus caudalis; scr, sulcus cranialis; v, ventriculus.

**FIGURE 3:**
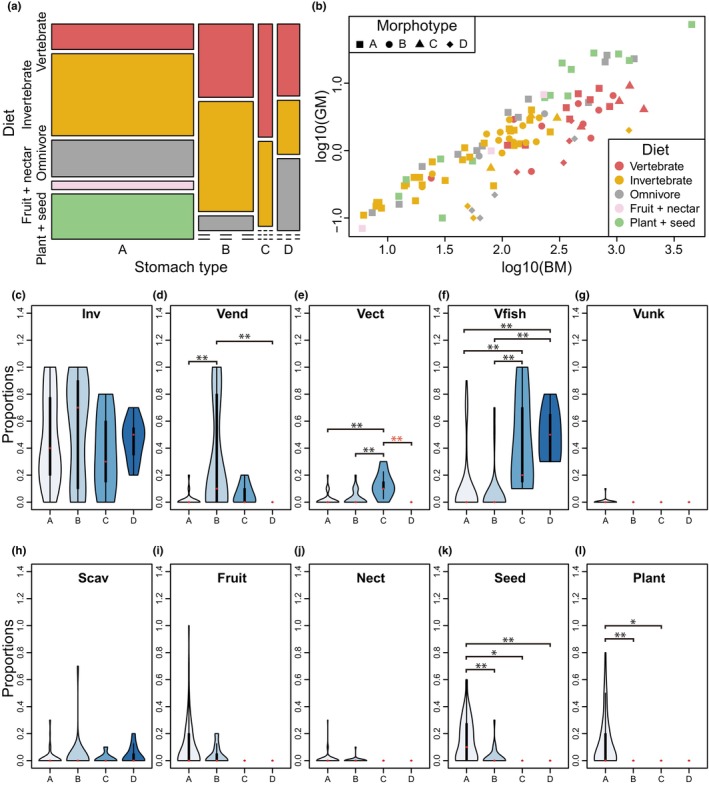
Graphical representations of diet–stomach morphotype–gizzard muscularity relationships. (a) A mosaic plot of diet against stomach type. (b) A scatter plot of ventriculus mass (VM) against body mass (BM). (c–l) Violin plots showing proportions of diet against stomach morphotype. The red dot represents the median value. The thick vertical line represents the interquartile range, and the thin vertical line represents the whisker range.

Through dissections and comparisons, we identified seven morphological characters across neornithine stomachs: ventriculus wall thickness, presence or absence of succus cranialis and succus caudalis, development of the gastric isthmus, ventriculus position, presence or absence of portio pylorica, proventriculus size, and centrum tendineum robustness (Figures 1 and 2, Figure S2). Using these morphological characters, a hierarchical cluster analysis differentiated neornithine stomachs into four clusters (Figure S1), designated as morphotypes A–D in this study (Figures 1, 2, and 3a–d). As described in detail later, there were intraspecific variations in the stomach morphological characters (Data S1), but the variation was small enough that there were no taxa showing more than one stomach morphotype.

Morphotype A stomach is represented by Anseriformes, Charadriiformes, Columbiformes, Galliformes, Gruiformes, Passeriformes, Podicipediformes, and Palaeognathae (Figure 1). The proventriculus is smaller than the ventriculus and is positioned anterior to the ventriculus in most taxa. The proventriculus wall is often thicker than the lumen and bears massive gastric glands in which the openings are macroscopically visible in large taxa. The junction between the proventriculus and the ventriculus is strongly constricted and forms a distinct gastric isthmus. The ventriculus is characterized by the well‐developed M. crassus cranioventralis and M. crassus caudodorsalis. These two large muscles are separated from small muscles M. tenuis craniodorsalis and M. tenuis cranioventralis by sulcus cranialis and sulcus caudalis, respectively. The deep incisions of sulcus cranialis and sulcus caudalis may provide extra space for storage when full and allow ventriculus contraction for mechanical processing of ingesta. The lateral sides of the ventriculus are covered with tendinous aponeuroses (centrum tendineum). An opening of the ventriculus for the duodenum is located left laterally at the ventral margin of the M. tenuis craniodorsalis. The large pyloric region between the ventriculus and the duodenum is short and is directly continuous with a deep groove of ventriculus lumen formed of two large longitudinal gastric folds and lacks isthmus that distinguishes the pyloric region from the ventriculus (Figure 1d). It should be noted that previous works reported the third stomach chamber portio pylorica from several members of Anseriformes and Gruiformes (Klasing, 1998; Ziswiler & Farner, 1972), which have a morphotype A stomach. Although this study agrees that some Anseriforms have a large pyloric region, this study does not treat it as a distinct stomach chamber due to the absence of a distinct isthmus on the ventriculus side (Figure 1d). It should also be emphasized that although the portio pylorica was previously referred to as a pyloric stomach (Ziswiler & Farner, 1972), it is not homologous with the pyloric stomach of Crocodylia, which is the closest group to Aves in extant vertebrates. Crocodylian pyloric stomach is instead homologous with avian ventriculus (Takasaki & Kobayashi, 2020b).

Morphotype A stomach is generally characterized by more muscular ventriculus than in the other stomach morphotypes. One exception is the diving duck *Mergus merganser*, which mainly feeds on fish. M. crassus cranioventralis and M. crassus caudodorsalis of *M. merganser* are reduced and form a stomach wall that is as thin as the proventriculus wall; therefore, the ventriculus is unlikely to serve as a site for mechanical processing. The difference in muscular wall thickness by diet is concordant with the intraspecific variation (Takasaki & Kobayashi, 2020a), although the situation in *Mergus merganser* is much more emphasized than the trend within a single species. Similarly, *Dromaius novaehollandiae* (Figure 1f, g) also shows a less muscular ventriculus, although it may be an artifact of the fact that the investigated specimen was derived from captivity. It should also be noted that a palaeognath *Struthio camelus* has a unique stomach among morphotype A. Although its ventriculus is of typical morphotype A, *S. camelus* is unique in anteroventral relocation of the ventriculus relative to the proventriculus (Figure 1h, i), as seen in morphotype D. Although the cause of this uniqueness is unknown, it should be kept in mind that ventriculus of *D. novaehollandiae* is another palaeognath in ordinary location, thus the status in *S. camelus* is unlikely a neornithine ancestral state. Note that despite the variations, *Dromaius*, *Struthio*, and *Mergus* are clustered with the other morphotype A taxa (Figure S1).

Morphotype B is represented by Accipitriformes, Camprimulgiformes, Coraciiformes, Cuculiformes, Falconiformes, Piciformes, and Strigiformes (Figure 2a–d). This type has a smaller proventriculus and more weakly constricted gastric isthmus than in morphotype A. The gastric wall of the ventriculus is also much thinner than that of morphotype A, although there are large variations: the gastric wall being thicker in *Picus awokera* and thinner in *Milvus migrans* (Figure 2c,d). The ventriculus of morphotype B differs from that of morphotype A in the absence of sulcus cranialis and sulcus caudalis (Figure 2a,b). Because sulcus cranialis and sulcus caudalis are absent, M. crassus and M. tenuis merge cranially and caudally; thus, the two muscles cannot be distinguished externally. The centrum tendineum of morphotype B is thin and less developed than in morphotype A and does not reach the caudodorsal or cranioventral margin of the ventriculus. The internal koilin layer is thin or absent. The opening for the duodenum is located on the anteroventral margin of the left lateral side of the ventriculus next to the opening for the proventriculus. The pyloric region does not form a distinct chamber (portio pylorica) in most specimens observed herein. The only exception is one specimen of *M. migrans* (HoUMVC 30,018), which has a distinct portio pylorica distinguished by two isthmuses in concordance with previous inferences (Klasing, 1998; Ziswiler & Farner, 1972). However, portio pylorica was not observed in another *M. migrans* specimen (HoUM 30112) as well as other members of Accipitriformes in this study, suggesting intraspecific variation of the structure.

Morphotype C is represented by Pelecaniformes and Suliformes (Figure 2e–j). This type has an enlarged proventriculus that is much larger than the ventriculus. The two chambers are externally indistinguishable due to the faint gastric isthmus. M. crassus is extremely reduced, resulting in a thinner gastric wall of the ventriculus than that of the proventriculus. Sulcus cranialis and sulcus caudalis are completely absent as in morphotype B, resulting in the union of M. crassus and M. tenuis. Centrum tendineum is extremely reduced and occupies less than 30% of the lateral surfaces of the ventriculus. Internally, the koilin layer is absent in all of the observed specimens, unlike the other stomach morphotypes. The pyloric region of the stomach is located on the anteroventral margin of the left lateral side of the ventriculus in Suliformes, whereas it is located on the anterodorsal margin of the ventriculus in Pelecaniformes. The pyloric region of morphotype C is unique in the presence of a third stomach chamber, portio pylorica, bordered from the ventriculus and the duodenum by two isthmuses, as previously reported in Ardeidae (Swenander, 1899; Ziswiler & Farner, 1972). The portio pylorica of *Ardea cinerea* is sphere‐shaped with a gastric wall as thick as the ventriculus. On the other hand, the portio pylorica of *Phalacrocorax carbo* is tubular as in *M. migrans* and internally bears longitudinal gastric folds.

Morphotype D is represented wholly by Procellariiformes (Figure 2k–n). Additionally, the stomach of *Eudyptes chrysocome* described by Jackson and Place (1990) suggests that sphenisciform is also likely to have the morphotype D stomach. The proventriculus of morphotype D is unique in its extreme longitudinal elongation, which is at least three times as long as the ventriculus. The posterior end of the proventriculus is recurved anteriorly. Therefore, the ventriculus of morphotype D is positioned ventrolateral to the proventriculus. A distinct gastric isthmus is present at the end of the anterior recurvation. The anterior recurvation and the resultant ventrolateral positioning of the ventriculus are unique to morphotype D among the observed specimens, except for *S. camelus*, as noted above. Although the ventriculus of morphotype D is small, its M. crassus cranioventralis and M. crassus caudodorsalis are more developed than all specimens of morphotype C, and some specimens of morphotype B. Sulcus cranialis and sulcus caudalis are completely absent, and the centrum tendineum is thin and undeveloped unlike in morphotype A. Internally, koilin layer is present (Figure 2n) in most observed individuals, although it is much thinner than the koilin layers of morphotypes A and B. The pyloric region is located at the posterodorsal margin of the right lateral side of the ventriculus. It should be noted that although the ventriculus is shifted, the position of the pyloric region is concordant with the other stomach types. The large pyloric region of the morphotype D does not have an extra isthmus in between the ventriculus and the pyloric region.

Fisher's exact test (two‐sided) suggests that the new stomach morphotypes proposed herein are associated with the neornithine diet categories previously defined (Wilman et al., 2014; *p* < 0.05; Figure 3a), including vertebrate, invertebrate, omnivorous, fruit + nectar, and plant + seed feeders. Morphotype A has the widest diet range, encompassing all categories, and is also the only morphotype that includes specialized herbivorous taxa (plant + seed and fruit + nectar feeders; Figure 3a, Table S1). Conversely, morphotypes B–D are specialized carnivores, mainly consuming vertebrates and invertebrates. The pairwise *t*‐tests on the relative dietary‐type abundance also support diet–stomach morphotype associations (Tables S2 and S3, Figure 3c–l). In summary, morphotype B consumes a larger proportion of endothermic animals than morphotypes A and D, morphotype C consumes a larger proportion of ectothermic animals than the other morphotypes, morphotypes C and D consume larger proportions of fish than morphotypes A and B, and morphotype A consumes larger proportions of seed and plants than the other morphotypes. At the same time, phylogenetic ANOVA shows that the diet–stomach morphotype association is not supported under the phylogenetic context other than the difference in the proportion of ectothermy feeding between morphotypes C and D (Table S3), although this does not necessarily negate the diet–stomach morphotype functional association.

The ventriculus mass is weakly negatively allometric in both GLS and PGLS models (Figure 3b, Table S4). High phylogenetic signals were detected for the diet category (lambda = 0.90, kappa = 0.15) and stomach morphotype (lambda = 1.00, kappa = 0.00), while gizzard muscularity is less phylogenetically constrained (lambda = 0.78, kappa = 0.06). In both GLS and PGLS, stomach morphotype had the most significant effect on the ventriculus mass (Table S4). Diet categories, on the other hand, showed no significant effect in either model, although they were slightly better fit than the prediction by the body mass alone. Proportions of several dietary types showed statistically significant effects under the GLS model (*p* < 0.05 for %Fish, %Fruit, %Seed, and %Plant). Under PGLS models, only %Fish and %Seed showed statistical significance, with fish being associated with a smaller, and seed with larger ventriculus mass. Note that the absence of significant correlations under PGLS models does not necessarily negate functional explanations, although it does not support convergent evolution.

## 4 DISCUSSION

This study categorized neornithine stomachs into four morphotypes based on a combination of seven characters (Figures S1 and S2). In addition to the four morphotypes shown here, Desselberger (1931) reported that multiple species of *Dicaeum* have a reduced ventriculus forming a diverticulum at the junction of the proventriculus and the duodenum. Additionally, *Opisthocomus hoazin* developed a foregut fermentation system using the crop and thus has an extremely reduced stomach (Grajal, 1995). While these birds suggest that the present categorization does not cover all of the neornithine stomach morphotypes in nature, this study covers most of the major extant avian orders and is the most comprehensive dataset at this point.

The categorization criteria for neornithine stomach morphotypes proposed herein align with previous works that associate avian stomach morphology with diets (Denbow, 2015; McLelland, 1979; Owen, 1866). Although the associations are not statistically significant under phylogenetically corrected models (Table S3), the associations are still likely to reflect the functional properties of each stomach morphotype. The thick stomach wall of morphotype A likely contributes to reducing the particle size of plant materials to access nutrients protected by cell walls (Moore, 1998), together with the aid of grits (e.g., Fritz et al., 2011), explaining its association with specialized plant + seed feeders. Morphotypes B, C, and D are primarily carnivorous but differ in their prey compositions. Morphotype B stomach generally feeds on terrestrial animals, whereas morphotypes C and D feed on fish and aquatic invertebrates. In these three morphotypes, the stomachs likely serve primarily as storage rather than sites for mechanical processing due to their large lumen in exchange for less muscular stomach walls. The large lumen facilitates the swallowing of a large chunk of meat, or even prey as a whole, benefiting in securing food.

While the diet–stomach morphotype association is evident, the series of phylogeny‐corrected analyses demonstrate strong phylogenetic constraints on neornithine stomach evolution. Similarly, diet had some effects on ventriculus mass under GLS, but the effects were insignificant under PGLS (Table S4). Although proportions of fish and seed feedings still retained statistical significance under PGLS models, even these two models have ΔAICc >10, suggesting that under the phylogenetic comparative framework, the effect of diet on neornithine ventriculus mass cannot be disentangled from that of phylogeny. The result differs from the intraspecific ventriculus mass flexibility associated with seasonal/experimental diet changes (e.g., Takasaki & Kobayashi, 2020a; van Gils et al., 2005). The difference shows that modifying the basic stomach architecture has a higher impact on ventriculus mass than the intraspecific variations.

Formally, these results negate the initial hypothesis that the stomach optimized for a specific diet over the course of avian evolutionary history. The strong constraints may be attributed to functional limitations. Although morphotype A allows for specialized herbivory in neornithines, their enlarged and heavy stomach contradicts the flight ecology of neornithines, which is established by numerous weight‐saving adaptations, including skeletal pneumatizations (Dumont, 2010). Therefore, the re‐acquisition of morphotype A, once lost in evolutionary history, might have been counter to selective pressure favoring enhanced flight ability. The possible exception to this pattern is Passeriformes, which independently acquired a modified morphotype A stomach from morphotype B. This exception may be related to their small body size and unique flap‐bounding flight ecology (Bruderer et al., 2010; Tobalske et al., 2009), which may thus have different selective pressures compared to other neornithine taxa (Orkney & Hedrick, 2024).

The constraints on morphotypes C and D may be linked to their aquatic dietary ecology, as previously suggested (Swenander, 1901). The portio pylorica in morphotype C and the shifted ventriculus in morphotype D are often filled with indigestible materials such as cephalopod beaks and gastroliths, and may function as a filter when consuming food with a large amount of water (Ziswiler & Farner, 1972). Furthermore, these stomach morphotypes have structural distinctions from the other morphotypes: morphotype C has an extra stomach chamber, and morphotype D dynamically shifts the ventriculus position. Therefore, functional limitations related to dietary ecology and structural distinctions might have contributed to the phylogenetic constraints observed in neornithine stomach morphotypes.

Our finding that stomach evolution does not closely correspond to the neornithine dietary ecology evolution courses is consistent with the recent finding that diet has negligible effects on neornithine intestine lengths (Duque‐Correa et al., 2022), unlike in mammals (Duque‐Correa et al., 2021). Duque‐Correa et al. (2022) further showed that flight mode may have a meaningful effect on intestine length. Although our study does not perform statistic analyses testing the flight mode–stomach relationship, the large ventriculus mass is unlikely beneficial for high flight performance. The present finding may thus further emphasize the impact of flight adaptation on forming the neornithine digestive tract, differing from mammals where diet has a larger contribution to forming digestive tract properties (Duque‐Correa et al., 2021).

## AUTHOR CONTRIBUTIONS

R.T. designed the project, performed dissections, wrote the paper, developed the methods, analyzed the data, and acquired the funding. Y. K. designed the project, supervised the research, and substantially edited the paper.

## FUNDING INFORMATION

This work is funded by the Grant‐in‐Aid for JSPS Research Fellow: 17J06410.

## CONFLICT OF INTEREST STATEMENT

The authors have no conflict of interest to declare.

### OPEN RESEARCH BADGES

This article has earned an Open Data badge for making publicly available the digitally‐shareable data necessary to reproduce the reported results. The data is available at [https://doi.org/10.6084/m9.figshare.25864786].

## CODE AVAILABILITY

The R script used for the analyses is provided as the Data S3.

## ETHICS STATEMENT

This article does not contain any studies with animals performed by any of the authors.

## Supporting information


Data S1.

## Data Availability

All data analyzed during this study are reposited in figshare (10.6084/m9.figshare.25864786).
