# Supplementary figures and images for: Anatomical description of neornithine stomach with implications on neornithine stomach morphology
Source: J Anat. 2024 Aug 17;245(5):787–96. doi: 10.1111/joa.14123 (PMC11470792; doi:10.1111/joa.14123)

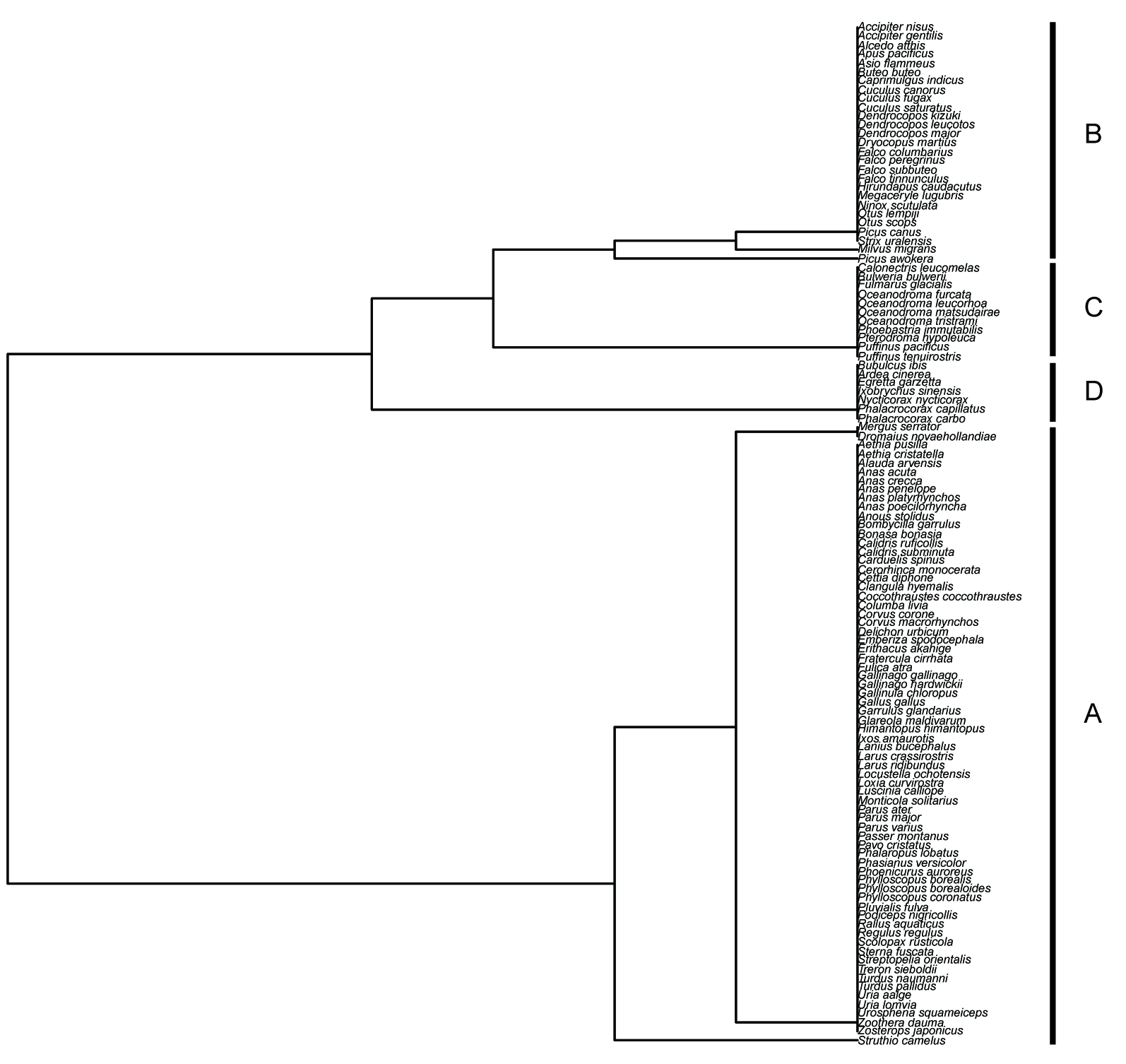

Supplement: Supplementary file 1 — Data S1. [file JOA-245-787-s001.zip › joa14123-sup-0004-FigureS1.tif]

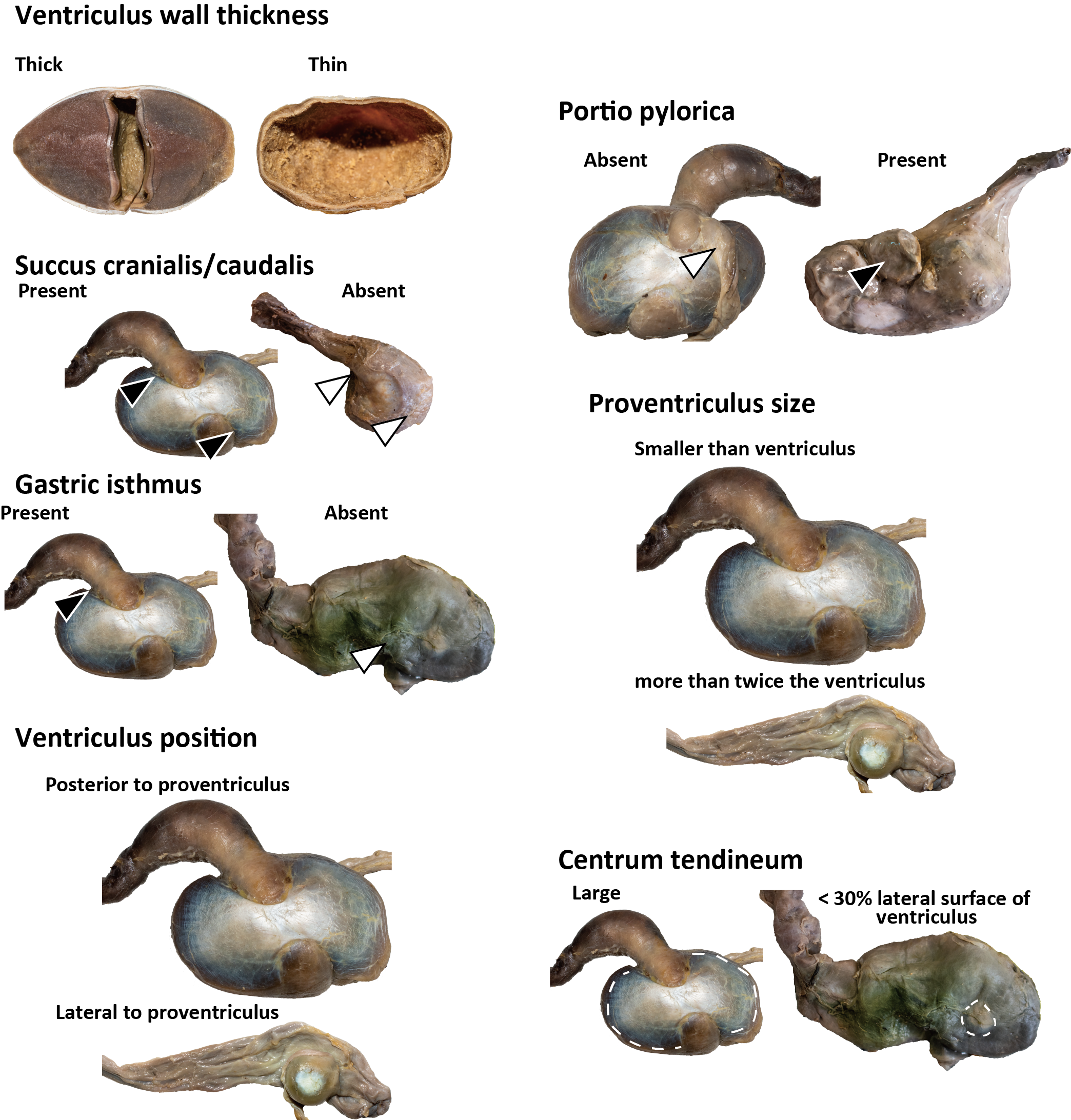

Supplement: Supplementary file 1 — Data S1. [file JOA-245-787-s001.zip › joa14123-sup-0005-FigureS2.tif]
